# Dosage strategies for delaying resistance emergence in heterogeneous tumors

**DOI:** 10.1002/2211-5463.13129

**Published:** 2021-03-30

**Authors:** Vahideh Vakil, Wade Trappe

**Affiliations:** ^1^ Wireless Information Network Laboratory (WINLAB) Rutgers, The State University of New Jersey New Brunswick NJ USA

**Keywords:** clonal evolution, containment, dosage strategy, drug resistance, optimal control

## Abstract

Drug resistance in cancer treatments is a frequent problem that, when it arises, leads to failure in therapeutic efforts. Tumor heterogeneity is the primary reason for resistance emergence and a precise treatment design that takes heterogeneity into account is required to postpone the rise of resistant subpopulations in the tumor environment. In this paper, we present a mathematical framework involving clonal evolution modeling of drug‐sensitive and drug‐resistant clones. Using our framework, we examine delaying the rise of resistance in heterogeneous tumors during control phase of therapy in a containment treatment approach. We apply pharmacokinetic/pharmacodynamic (PKPD) modeling and show that dosage strategies can be designed to control the resistant subpopulation. Our results show that the drug dosage and schedule determine the relative dynamics of sensitive and resistant clones. We present an optimal control problem that finds the dosing strategy that maximizes the delay in resistance emergence for a given period of containment treatment.

## Graphical abstract

We present a mathematical framework involving clonal evolution modeling of drug‐sensitive and drug‐resistant clones to examine delaying the rise of resistance in heterogeneous tumors during the control phase of therapy in a containment treatment approach. We propose an optimal control problem that finds the dosing strategy that maximizes the delay in resistance emergence, in the tumor environment.

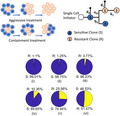

**Abbreviations**
PKPD: pharmacokinetic/pharmacodynamic
MTD: maximum tolerated dose
MIC: minimum inhibiting concentration

## Introduction

The emergence of resistance is a major challenge and one of the main barriers to the success of cancer treatment strategies. The microenvironment associated with a tumor, as well as its heterogeneity, is the leading contributor to cancer relapse because they foster treatment resistance. Drug resistance in cancers is the ability of a group of cells within a tumor to survive a therapy due to a genetic alteration or new mutations. Tumor evolution and the rise of multiple subpopulations of cancer cells, each with a potentially different response to therapeutics, play a key role in tumor drug resistance.

Different treatment strategies have been explored to combat the emergence of resistance. Combination therapies are a leading approach toward this goal [1, 2]. However, conventional treatment methods that are based on the continuous administration of fixed dosages of single or multiple drugs have been shown to be less effective than treatment methods that consider the current state of the tumor. While using maximum tolerated dose (MTD) is thought to achieve maximum therapeutic benefit by killing a large number of cancerous cells, there is increasing evidence that undermines this approach and the basis for its application [3]. The authors in [4] propose an optimization problem for determining a dosage strategy in combination therapy to combat drug resistance in tumor progression. The authors in [3] introduce a submaximal therapeutic protocol that is designed based on the complex trade‐offs between the death of cancerous cells, the emergence of resistance, and metastases. They provide evidence that a more personalized treatment, which integrates patient‐specific evolutionary dynamics, would be more effective than the MTD regimen. The effectiveness of administering high dosages for a long period of time as an aggressive treatment versus a moderate strategy with low dosages in short duration has been compared through empirical studies [5]. In [6], the authors propose an adaptive therapy that is optimized based on an evolutionary game theoretic model for cancer dynamics. The method presented optimizes the total drug usage and time to recovery and is shown to outperform the standard treatment, which is based on a continuous use of maximum tolerated doses. The authors in [7] present a treatment method for metastatic castrate‐resistant prostate cancer based on a multidrug adaptive therapy. They illustrate the tumor evolution through frequency‐dependent cycles that lead the tumor into a controllable loop and provide a path for repeatable multidrug adaptive therapy. The authors in [8] investigate cancer treatment as a contest between the treatment and resistance strategies and present a game theoretic approach for designing successful treatment protocols. They argue that by constantly administering the same drugs or by changing the treatment only in response to tumor progression, treatment failure would be inevitable. However, by integrating the evolutionary dynamics into the treatment protocols and exploiting adaptive therapeutic methods, the outcome can be improved.

To design effective treatment protocols that manage resistance [9] considers the effect of different therapeutic methods on the fitness of resistant and sensitive types. The authors suggest that competitive release, defined as the increase in absolute fitness of a resistant clone and which occurs when the sensitive type is removed by therapy, enhances the probability and rate of resistance emergence. In other words, the treatment methods that aim at eliminating the drug‐sensitive subpopulations lead to tumor microenvironment alteration that favors the drug‐resistant subpopulation [10]. Eradicating the sensitive clone results in a rapid, unchecked growth of the resistant subpopulation and a reduction in heterogeneity toward forming an entirely resistant tumor.

**Fig. 1:**
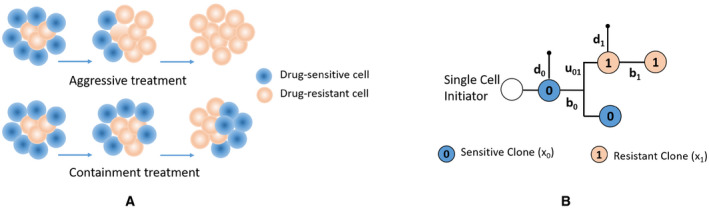
(A) In aggressive treatments, eradicating the drug‐sensitive cells leads to a rapid growth of resistant cells and forming an entirely resistant tumor. However, containment treatments maintain competition between the subpopulations, thereby preventing resistant cells from taking over the entire tumor. (B) Basic model of clonal evolution for one original cell and one mutation. The birth and death rates of cell m are represented by $b_{m}$ and $d_{m}$, respectively, and the mutation rate from type 0 to type 1 is denoted by $u_{01}$

On the other hand, a natural force that can suppress or delay the rise of resistance is the competition between sensitive and resistant subpopulations, such as for nutrient resources [10]. Therefore, when the size of a tumor is tolerable but it is rapidly mutating, a containment treatment strategy is advantageous as it allows the sensitive clonal population to survive and compete with the resistant subpopulation [10, 11, 12]. The purpose of such a treatment is to keep the tumor at the tolerable burden and to delay the emergence of resistance. For this method, the goal is not to eradicate the whole population of sensitive cells that respond to chemotherapy, but instead keep a portion of this population alive to compete with drug‐resistant cells and keep them from reproducing unchecked, taking over the entire tumor and thereby leading to a competitive release that could have more dire consequences for the patient. Figure 1A graphically compares the aggressive and containment treatment strategies. In the case that the patient immune response increases over time, delaying the emergence of resistance might provide sufficient time for immunity to help in preventing the resistance [11].

Delaying the rise of resistance is an important outcome of the containment treatment strategy in various ways: (i) it could be used as a factor for monitoring the treatment effectiveness; (ii) it can provide some additional time for the immune system to recover; and (iii) it can provide sufficient time for incorporating other therapeutic methods (such as immunotherapy) in a polytherapy approach. Designing a proper dosage strategy that maintains the balance between sensitive and resistant subpopulations while avoiding toxicity is required for combating or delaying resistance. In this paper, we provide a mathematical framework that captures the competitive dynamics of the resistant and sensitive subpopulations within a tumor. Then, through pharmacokinetic/pharmacodynamic (PKPD) modeling, we explore the effect of dosage strategies on the relative dynamics of these subpopulations. Based on this model, we propose an optimization problem that finds the optimal dosing that maximizes the emergence time of the resistant clonal population.

The rest of the paper is organized as follows. In section Methods, we present the mathematical framework for competition dynamics in a clonal evolution model. Additionally, we model the dynamic and kinetic effects of the drug in a containment treatment strategy. Then, we propose our optimal solution to determine the dosage strategy that leads to the latest emergence of the resistant clone. In section Results and Discussion, we provide the results of our framework and our proposed optimal solution. In section Conclusion, we conclude the paper.

## Methods

In a heterogeneous environment, where all subpopulations compete over natural resources and nutrients, mathematical modeling is a pivotal approach to exploring the dynamics of the competing clones. In this section, we overview some works in the literature that model the dynamics of competitive subpopulations. Afterward, we present the mathematical model that we employ throughout this paper for exploring the competition between drug‐resistant and drug‐sensitive cells in a heterogeneous tumor.

### Related work: competition dynamics

In [13], the authors model the dynamics of competing cancer clones through an approach similar to the competition among different species in ecology. In a two‐species model, they present the following system of equations

(1a) $$\dot{x}=r_{x}x\left(1‐\frac{x+\gamma_{\mathrm{xy}}y}{K}\right),$$

(1b) $$\dot{y}=r_{y}y\left(1‐\frac{y+\gamma_{\mathrm{yx}}x}{K}\right),$$

where *K* represents the carrying capacity as the maximum size possible for the cell population. *r_x_* and *r_y_* are the growth rates of species *x* and *y*, respectively. In this classic model, *ϒ_xy_* and *ϒ_yx_* represent the degree of interspecific competition, defined as the amount of each subpopulation's effect on the growth of the other one. In the case that there is no interaction between the species or there is only one type, (1) reduces to a Logistic growth model for each species. Although there is no closed‐form solution for the system (1), [13] supports the interpretation of long‐term behavior through linear stability analysis. The authors note that the steady‐state dynamics of the species leads to competitive exclusion or coexistence, depending on the system parameters. The authors show that both species coexist in the long‐term, if and only if each of them suppresses its own type more than its competitor. Under the opposite condition, only one of the species would survive in the long‐term, a situation that is called competitive exclusion.

Other papers, such as [14, 15], follow a similar approach to extending the model for expressing the evolutionary dynamics of multiple cell types and the competition of resistant and sensitive types in a competitive heterogeneous tumor environment. Following a different approach, [16] focuses on the dynamics of resistant cells given a generic growth model for the entire population. Then, for Gompertz, Logistic, and exponential dynamics, the authors study the effect of aggressive chemotherapy on the size of the resistant population. Following the equation presented in [16] for logistic growth, the authors in [11] propose a mathematical framework to determine the density of the drug‐resistant population in infective diseases. The authors employ this model to express the benefits and costs for the sensitive population through competition and mutation, and present a managed treatment strategy including aggressive and containment strategies. For an antibiotic setting, the authors in [12, 17] define a resource‐dependent reproduction rate to determine the effective treatment strategy in the presence of competition between antibiotic‐resistant and antibiotic‐sensitive pathogens. They assume that the drug affects both populations but with different effectiveness as represented by the minimum inhibiting concentration (MIC) of the antibiotic. The results presented in [12] show that the competition between resistant and sensitive cells determines whether an aggressive or moderate treatment strategy minimizes the resistance.

### Resource‐constrained modeling of clonal evolution

In this paper, we consider a basic clonal evolution model, following Nowell's branching architecture [18], as represented in Fig. 1B for an original cell and its mutation. Table 1 contains all of the parameters and variables that will be used in our model and solutions for the rest of the paper. The birth rate and death rate are represented by $b_{m}$ and $d_{m}$ for cell m, respectively, and the rate of mutation from cell type 0 to 1 is denoted by $u_{01}$. These cell types represent the drug‐sensitive (with size $x_{0}\left(t\right)$) and drug‐resistant (with size $x_{1}\left(t\right)$) clones. Associated with this model, we derive the set of equations (2) that express the dynamic behavior of the clonal evolution. In order to explore the competition among the resistant and sensitive clones over nutrients, similar to [12], we introduce a nutrient source $R\left(t\right)$ which varies proportional to the growth rates $G_{0}$ and $G_{1}$ of the clones. However, unlike [12], we assume that the present populations are the only factors that affect the nutrient resource supply.

(2a) $${\dot{x}}_{0}=b_{0}x_{0}‐d_{0}x_{0}‐u_{01}x_{0},$$

(2b) $${\dot{x}}_{1}=b_{1}x_{1}‐d_{1}x_{1}+u_{01}x_{0},$$

(2c) $$\dot{R}=‐\alpha \left(G_{0}x_{0}+G_{1}x_{1}\right),$$

where the time variable t is dropped for the sake of simplicity, $G_{0}=b_{0}‐d_{0}$ and $G_{1}=b_{1}‐d_{1}$.

**Table 1:** Parameters and variables

Notation	Description
*x_0_*	Size of sensitive type
*x_1_*	Size of resistant type
*b_m_*	Birth rate of cell type m
*d_m_*	Death rate of cell type m
*u*_01_	Mutation rate from type 0 to type 1
*R*	Nutrient resource
*α*	Rate of nutrient resource usage
*R_0_*	Nutrient related constant at initiation
λ*_m_*	Birth rate–nutrient factor for type m
*f*_PD_(.)	Pharmacodynamics (PD) model
*f*_PK_(.)	Pharmacokinetic (PK) model
*c_p_*	Plasma concentration of a drug
*κ*	Decay rate for a drug
*E*_max_	Maximum response of a drug
*EC*_50_	Half maximum concentration of a drug
*K*_lm_	Linear approximation factor on type m
$h_{m}^{t}$	PKPD effect of drug on type m
*N*	Number of doses
*T_n_*	Drug's *n* ^th^ administering time instant
$D_{n}$	Dosage at *n* ^th^ time instant
$X_{0}$	Vector of the initial number of cells
$t_{0}$	Time of initiation
$t_{\mathrm{eq}}$	Time of resistance emergence
$t_{f}$	Length of the treatment period
$\tau_{\min}$	Minimum dosage interval
$D_{\min}$	Minimum dosage of drug
$D_{\max}$	Maximum dosage of drug

From the above equations, it is concluded that $\dot{R}=‐\alpha \left({\dot{x}}_{0}+{\dot{x}}_{1}\right)$, which means $R+\alpha \left(x_{0}+x_{1}\right)=R_{0}$, where $R_{0}$ is a constant defined at the time of initiation ($t_{0}$) as $R_{0}=R\left(t_{0}\right)+\alpha \left(x_{0}\left(t_{0}\right)+x_{1}\left(t_{0}\right)\right)$. Adopting the linear approximation for the birth rate–nutrition relationship, we can express $b_{m}$ as a linear function of the available resource as $b_{m}=\lambda_{m}R$. Therefore, given that $R=R_{0}‐\alpha \left(x_{0}+x_{1}\right)$,

(3) $$b_{m}=\lambda_{m}\left(R_{0}‐\alpha \left(x_{0}+x_{1}\right)\right),m=0,1.$$

The system of (2), then reduces to a system of two equations that represent the dynamics between the competing clones as

(4a) $${\dot{x}}_{0}=\lambda_{0}\left(R_{0}‐\alpha \left(x_{0}+x_{1}\right)\right)x_{0}‐d_{0}x_{0}‐u_{01}x_{0},$$

(4b) $${\dot{x}}_{1}=\lambda_{1}\left(R_{0}‐\alpha \left(x_{0}+x_{1}\right)\right)x_{1}‐d_{1}x_{1}+u_{01}x_{0}.$$

Factoring $R_{0}$ in the above equations leads to

(5a) $${\dot{x}}_{0}=\lambda_{0}R_{0}\left(1‐\frac{\left(x_{0}+x_{1}\right)}{R_{0}/\alpha }\right)x_{0}‐d_{0}x_{0}‐u_{01}x_{0}$$

(5b) $${\dot{x}}_{1}=\lambda_{1}R_{0}\left(1‐\frac{\left(x_{0}+x_{1}\right)}{R_{0}/\alpha }\right)x_{1}‐d_{1}x_{1}+u_{01}x_{0},$$

meaning that for a total cell quantity greater than $R_{0}/\alpha$, the variation rate would be negative.

### Optimal dosage for containment in heterogeneous tumors

In this paper, it is assumed that that an aggressive treatment has been applied to reduce the size of tumor to a tolerable burden with a rationale similar to the method proposed in [4]. Then, in the control phase of the treatment, a containment strategy is followed to delay resistance emergence. In this section, we present a mathematical framework to explore the relative dynamics of sensitive and resistant subpopulations in a containment strategy and to maximize the time for resistance emergence through PKPD modeling of the administered drug.

In this phase, we introduce a drug that is effective on sensitive cells but has very small or no effectiveness on the resistant clone. Assuming that the effect of a drug on killing tumor cells follows first‐order kinetics [19], the system of equations (2) is then modified as

(6a) $${\dot{x}}_{0}=b_{0}x_{0}‐d_{0}x_{0}‐u_{01}x_{0}‐h_{0}^{t}x_{0}$$

(6b) $${\dot{x}}_{1}=b_{1}x_{1}‐d_{1}x_{1}+u_{01}x_{0}‐h_{1}^{t}x_{1}$$

(6c) $$\dot{R}=‐\alpha \left(G_{0}x_{0}+G_{1}x_{1}\right)$$

with $G_{0}=b_{0}‐d_{0}‐h_{0}^{t}$ and $G_{1}=b_{1}‐d_{1}‐h_{1}^{t}$. The time‐variant effect of the drug on the sensitive and resistant clones is represented by $h_{0}^{t}$ and $h_{1}^{t}$, respectively. The superscript t is used to distinguish these time‐varying functions from the constant coefficients for birth, death, and mutation rates.

Assuming that plasma concentration—which is related to pharmacokinetic parameters—and the concentration at the site of action—which is present in pharmacodynamic models—are always in equilibrium, the effect of the drugs can be modeled using PKPD modeling [20]. Therefore, $h_{m}^{t}$ can be expressed for the drug response time period as $h_{m}^{t}=f_{\text{PD}}\left(f_{\text{PK}}\left(\text{dose},t\right)\right)$, where $f_{\text{PD}}\left(.\right)$ is the pharmacodynamics model and $f_{\text{PK}}\left(.\right)=c_{p}\left(t\right)$ is the pharmacokinetic model in which $c_{p}\left(t\right)$ is the time‐variant plasma concentration [20]. The pharmacokinetic model for drugs that are administered intravenously follows exponential decay as

(7) $$c_{p}\left(t\right)=c_{p_{0}}e^{‐\kappa t},$$

where $c_{p_{0}}$ is the initial plasma concentration at the time of administration and κ is the decay rate and depends on drug clearance. The effective response of a drug with concentration c, follows the empirical pharmacodynamic model

(8) $$E\left(c\right)=\frac{E_{\text{max}}c^{\nu }}{{\text{EC}}_{50}^{\nu }+c^{\nu }},$$

where c is the concentration of the drug, and ${\text{EC}}_{50}$ is the concentration that produces half of the drug's maximum response ($E_{\text{max}}$) and ν is a Hill‐type factor governing the sigmoidicity of the response. The linear adaptation of (8) approximates this model for small concentration values [20] as $E\left(c\right)\approx \frac{E_{\text{max}}}{{\text{EC}}_{50}}c=K_{l}c$, where $K_{l}$ is called the linear approximation factor, in this paper.

Therefore, $h_{0}^{t}=K_{l0}c\left(t\right)$ and $h_{1}^{t}=K_{l1}c\left(t\right)$, where $K_{l1}\ll K_{l0}$ given that cell type 1 is resistant to the drug. Assume that at time instants ${\left\{T_{n}\right\}}_{n=1}^{N}$, where N is the total number of doses, a new dose $D_{n}$ of drug is taken. The plasma concentration at the beginning of each new administration is expressed as

(9) $$c_{p_{n}}=D_{n}+c_{p_{n‐1}}e^{‐\kappa \left(T_{n}‐T_{n‐1}\right)},$$

where the second term reflects the remaining effect of the previous dosage.

It can be considered that in the system of equations (6), $\dot{R}=‐\alpha \left({\dot{x}}_{0}+{\dot{x}}_{1}\right)$. Following the rationale that led to representing $b_{m}$ as in equation (3), the system of equations (6) can be expressed in the form of

(10a) $${\dot{x}}_{0}=\lambda_{0}\left(R_{0}‐\alpha \left(x_{0}+x_{1}\right)\right)x_{0}‐d_{0}x_{0}‐u_{01}x_{0}‐h_{0}^{t}x_{0}$$

(10b) $${\dot{x}}_{1}=\lambda_{1}\left(R_{0}‐\alpha \left(x_{0}+x_{1}\right)\right)x_{1}‐d_{1}x_{1}+u_{01}x_{0}‐h_{1}^{t}x_{1},$$

or equivalently as

(11) $$\frac{dX}{dt}=f\left(t,X\left(t\right),D,T\right).$$

The N‐dimensional real vector D contains all required dosages. T is an N‐dimensional vector with positive integer elements that contains all time instants and X is an M‐dimensional vector that represents the size of all cell types. In the case of (10), M=2 and $X={\left[x_{0}x_{1}\right]}^{T}$. There is no general closed‐form expression for the solution of this nonlinear system, though we can analyze the response for specific conditions.

In the case that the treatment is able to completely eradicate the sensitive clone, the number of cells in this subpopulation would be $x_{0}=0$. Assuming $h_{1}^{t}$ is negligible, at the steady‐state $\frac{dX}{dt}=0$, the number of resistant clone cells would be $x_{1}=\frac{\lambda_{1}R_{0}‐d_{1}}{\lambda_{1}\alpha }$ and can be approximated by $R_{0}/\alpha$, which equals the maximum size of the tumor, in the control phase. In other words, eliminating the sensitive cells leads to a tumor that is entirely formed by the resistant clone $x_{1}$ and thus changes the tumor cell population in favor of the resistant clone.

In order to prevent this tumor environment alteration, we adopt a containment treatment strategy and propose a dosage design that delays the rise of the resistant clone over a fixed, known period of time. We use the time instant at which the size of the resistant subpopulation surpasses the sensitive subpopulation as the indicator of resistance emergence. In other words, this is the time instant that each clone equally forms half of the tumor and is shown by $t_{\text{eq}}$ in this paper. The following optimization problem finds the optimal dosage strategy that maximizes the time for resistance emergence or equivalently maximizes $t_{\text{eq}}$.

(12a) $$\underset{D,T}{max}t_{\text{eq}}$$

(12b) $$\text{Subject to}\;\frac{dX}{dt}=f\left(t,X\left(t\right),D,T\right)$$

(12c) $$X\left(t_{0}\right)=X_{0}$$

(12d) $$x_{1}\left(t_{\text{eq}}\right)=x_{0}\left(t_{\text{eq}}\right)$$

(12e) $$T_{1}\geq t_{0}+\tau_{\text{min}}$$

(12f) $$t_{f}\geq T_{N}+\tau_{\text{min}}$$

(12g) $$T_{n}\geq T_{n‐1}+\tau_{\text{min}},2\leq n\leq N$$

(12h) $$D_{\min}\leq D_{n}\leq D_{\max},1\leq n\leq N$$

where $t_{f}$ is the treatment period; $D_{n}$ and $T_{n}$ are the $n^{\mathrm{th}}$ dosage and time instant, respectively. $\tau_{\min}$ is the minimum time between two consecutive courses; $D_{\min}$ and $D_{\max}$ are the drug's minimum and maximum dosages, respectively.

## Results and Discussion

In this section, we provide a quantitative formulation and mathematical framework that models the effect of drugs on the clonal evolution of competing cells, and the impact of therapeutics on delaying the emergence of resistance during the control phase. Table 2 contains the baseline values of the input parameters assumed in this evaluation which are mainly extracted from [4, 13, 21]. The table also shows the range of the parameters that will be used in the sensitivity analysis of the model. In our evaluations, we assume that the number of sensitive cells at the beginning of control phase is $10^{3}$ and that there exists a very small number of resistant cells, set to 10. Other parameters are assumed to be equal to the baseline values reported in Table 2.

**Table 2:** Input parameters values and range

Parameter	Value^a^ and Range^b^	Unit
$\lambda_{0}$	0.185±10%	day^−1^/µg
$\lambda_{1}$	0.12±10%	day^−1^/µg
$d_{0}$	0.1±10%	day^−1^
$d_{1}$	0.02±10%	day^−1^
$u_{01}$	$10^{‐4}\pm 10\text{\%}\;$	day^−1^
$R_{0}$	1500	µg
α	0.9±10%	µg/cell
$K_{l0}$	2±10%	day^−1^/(µm/l)
$K_{l1}$	0.125±10%	day^−1^/(µm/l)
κ	0.0857±10%	day^−1^
N	4	–
$t_{f}$	30	day
$\tau_{\min}$	4	day
$D_{\min}$	0.05	µm/l
$D_{\max}$	0.5	µm/l

^a^ Baseline values were extracted from [4, 13, 21].

^b^ Range defined for the sensitivity analysis.

**Fig. 2:**
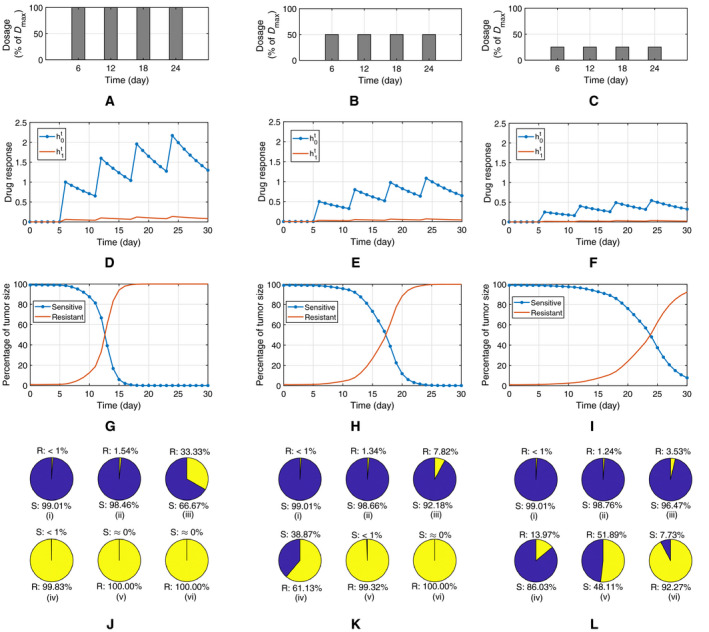
The effect of drug dosage on the clones' dynamics in a containment treatment for a period of 30 days; (A–C) three different dosage schedules; (D–F) the associated drug response ($h_{0}^{t}$ and $h_{1}^{t}$) to the schedules (A), (B) and (C); (G–I) percentage of tumor size for drug‐sensitive and drug‐resistant clones over the period of treatment; J–L percentage of sensitive (S) and resistant (R) cells at six check points from (i) to (vi), including the initial, after each administered dose and at the final point. From the results, it is apparent that the relative dynamics of the subclones and time of rising the resistance depend on the dosage schedule

In Fig. 2, we explore the effect of the dosage on the emergence of resistance by lowering the dosage amount following the schedules presented in graphs (A)–(C), from a full amount of $D_{\max}$ to 50% and 25% of its baseline value. This leads to decreasing the drug response ($h_{0}^{t}$ and $h_{1}^{t}$), as depicted in graphs (D)–(F). The number of cells in the sensitive and resistant subpopulation is shown in (G)–(I), in terms of the percentage of the tumor's size. The graphs show that when we reduce the drug dose, the competition between the drug‐sensitive and drug‐resistant clones leads to delaying the emergence of resistance. In graphs (J)–(L) the size of sensitive and resistant clones is compared at six different checkpoints, for each of the dosing schedules (A)–(C) explored in this figure. The checkpoints include the initial time for the control phase, four time instants after each of the four drug administrations and at the end of treatment period. It is apparent that the size of resistant clone increases after each dose. More importantly though, these graphs show that in plot (J), which is associated with the dosing schedule (A), the resistant type takes over the entire tumor after the third administration, while it happens after the fourth dosage in (K) and it is the dominant subpopulation by the end of the treatment in (L). From the results presented in this figure, it is concluded that the relative dynamics and population for the drug‐sensitive and drug‐resistant clones are governed by their competition and depend on the dosing strategy.

**Fig. 3:**
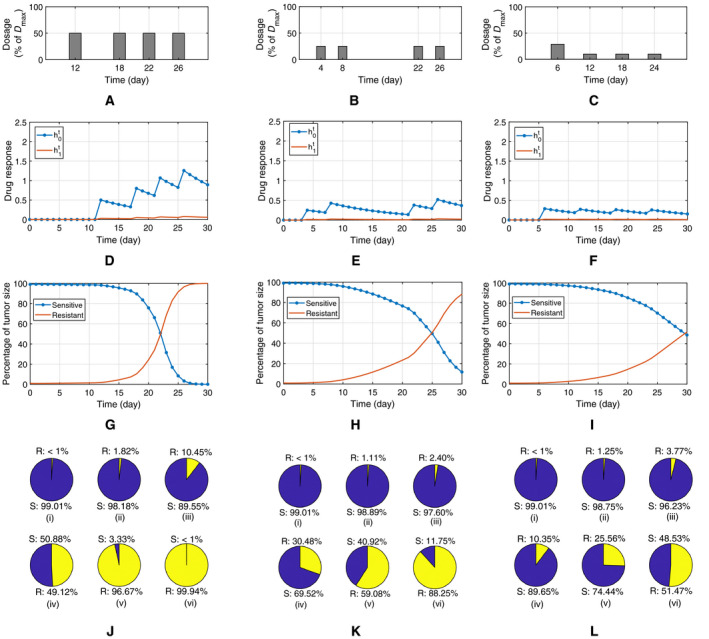
The effect of drug dosage on the clones' dynamics in a containment treatment for a period of 30 days and delaying the emergence of the resistant clone through optimal scheduling; (A) and (B) two different dosage schedules and (C) is the optimal schedule found as the solution of the optimization problem; (D–F) the associated drug response ($h_{0}^{t}$ and $h_{1}^{t}$) to the schedules (A), (B), and (C); (G–I) Percentage of tumor size for drug‐sensitive and drug‐resistant clones over the period of treatment where (I) represents the optimal solution; (J–L) percentage of sensitive (S) and resistant (R) cells at six check points from (i) to (vi), including initial, after each dose of administration and at the final point. It is apparent that the relative dynamics of the subclones and the time of rising resistance depend on the dosage schedule and that our optimized strategy leads to delaying the emergence of the resistant clone

In the next set of investigations in Fig. 3, we present the effect of two other dosage strategies given in (A) and (B) in comparison with our proposed optimal strategy in (C) which is the solution of the optimization problem (12). The dosage values in (A) and (B) are 50% and 25% of $D_{\min}$, respectively, with different timings given in the graphs. The associated drug responses are presented in graphs (D)–(F), and the resultant sizes of drug‐resistant and drug‐sensitive clones are depicted in (G)–(I), where (I) represents the optimal solution. It is observed that in (I), the two curves representing the two subpopulations meet at the maximum possible time instant in the given treatment period, thus showing a delay in resistance onset. In plots (J)–(L), the percentage of resistant and sensitive subpopulations is compared at six checkpoints, which include the initial time instant, the time after four doses and at the end of the treatment period. It is apparent from (J) and (K) that in their corresponding strategies, (A) and (B), the resistant clone outcompetes the sensitive clone after the fourth dose. In the optimal strategy however, as shown in (L), the emergence of resistance is postponed so that, at the end of the treatment period, the size of resistant and sensitive clones are comparable. The results presented in this figure confirm that the relative dynamics of the sensitive and resistant clones is dependent on the dosing strategy. It is also concluded from Figs 2 and 3 that the proposed optimal dosing strategy in 3(c), which is the solution of the optimization problem, leads to the maximum delay in the onset of resistance.

**Fig. 4:**
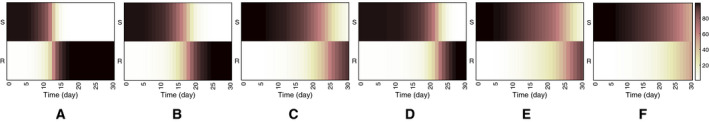
Heatmap plots displaying the amount of sensitive (S) and resistant (R) cells over the period of treatment. Heatmaps (A–C) correspond to dosage schedules explored in Figs 2A–C, plots (D) and (E) correspond to dosages in Figs 3A, Band map (F) represents the optimal strategy 3(C) where the size of sensitive and resistant subclones become equal at the end of the treatment period

Figure 4 displays the heatmap plots for the number of sensitive and resistant cells over the period of treatment for all of the dosage strategies given in Figs 2A–C and 3A–C, in the order of appearance. Plot 4(F), which is associated with the optimal dosage strategy, shows that the maximum size for the resistant clone population is achieved at the end of the treatment period, where it equals the size of sensitive clone. However, as depicted in 4(A)–(E), this crossing happens at distinct time instants depending on the dosage strategy.

**Fig. 5:**
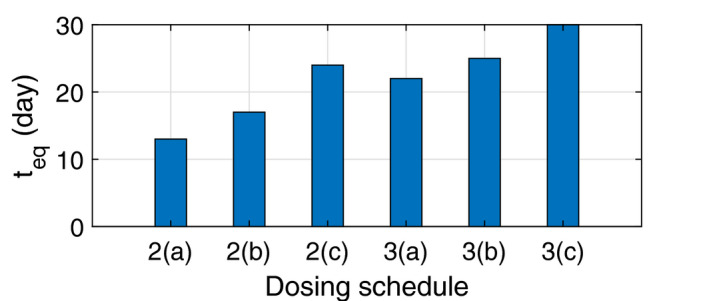
Delaying the emergence of the resistant clone through containment treatment. Comparing the effect of the dosing schedule on $t_{\mathrm{eq}}$, the time instant at which the size of resistant clone surpasses the sensitive population, for different schedules investigated in Figs 2 and 3. The bar labeled as 3(C) is associated with the optimal schedule, where $t_{\mathrm{eq}}$ reaches its maximum which equals the treatment period

Figure 5 compares $t_{\mathrm{eq}}$, the time instant that the size of the resistant subpopulation equals the size of sensitive clonal population, for the different dosage strategies given in Figs 2A–C and 3A–C. The plot confirms that changing the dosage strategy changes the time instant at which the resistant population surpasses the sensitive population and that the optimal strategy maximizes this time.

### Sensitivity analysis

**Fig. 6:**
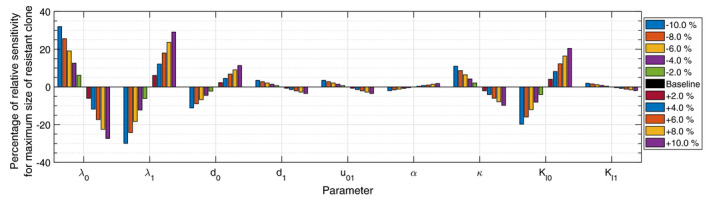
Percentage of relative sensitivity of the size of the resistant clone at the end of the treatment period to the model parameters as well as the drug's PKPD parameters. Each individual parameter was investigated independently, varying in a range of ‐10% to +10% of its baseline quantity as reported in Table 2, while the optimal dosage strategy, as in Fig. 3C, was assumed

We perform single parameter sensitivity analysis for the optimal dosage strategy and investigate the effect of the model parameters as well as drug's PKPD parameters on the maximum size of resistant clone that achieved at the end of the treatment period. In Fig. 6, the percentage of relative sensitivity is presented. While the optimal dosage strategy 3(c) assumed with the baseline values for the parameters, as listed in Table 2, one parameter at a time has been varied in a range of ‐10% to +10% of its baseline quantity. It is apparent in the figure that among the model parameters, the resistant clone population is most sensitive to the birth rate–nutrient parameters $\lambda_{0}$ and $\lambda_{1}$. Additionally, between drugs' PKPD parameters κ, $K_{l0}$, and $K_{l1}$, the size of the resistant clone is most sensitive to the drug's efficacy parameter on the sensitive clone, reflected by linear approximation factor $K_{l0}$.

## Conclusion

In thCis paper, we proposed a mathematical framework for modeling the competition between drug‐sensitive and drug‐resistant populations through clonal evolution dynamics of heterogeneous tumors. The framework involves a PKPD model for the administered drug, which defines the relative dynamics of the competing clones. It was shown that, for a containment therapy, the control phase of the treatment (which aims to control and maintain the tumor burden at a tolerable level) can be mathematically formulated to achieve a strategically designed dosing strategy that will lead to the postponement of resistance emergence. To support more effective containment strategies, we formulated an optimization problem that determines the dosage strategy that leads to the latest onset of resistance for a given treatment period. We presented simulation results that support the validity of the proposed methodology and the optimization algorithm. We compared the relative percentage of the tumor's size for the sensitive and resistant cells over the course of the treatment. Additionally, we compared the size of the sensitive and resistant clonal populations at different checkpoints, including the initiation time for the control phase, after the administering of each dose and at the end of the treatment period. Our results showed that, while the resistant clonal population increases after each dosage, the respective fraction of each clone is governed by the presence of interclonal competition. We investigated the effect of the dosage strategy on the relative dynamics of the sensitive and resistant subpopulations by altering the dosage amount, as well as the administration schedule. Our results showed that with a reduction of the drug dose from the standard, maximum dose of $D_{\max}$, the competition between the drug‐sensitive and drug‐resistant clones leads to delaying the emergence of resistance and that changing the dosing schedule results in different resistance dynamics. Notably, our proposed optimized dosage strategy increased the time of resistance emergence by more than 100%, with respect to the dosing scenario with the baseline value of $D_{\max}$. Single parameter sensitivity analysis showed that, under optimal dosage strategy, the size of resistant clone population is more sensitive to the birth rate–nutrient parameters and the drug's efficacy on the sensitive clonal population than it is to the dynamics associated with population death and mutation rates.

## Conflict of interest

The authors declare no conflict of interest.

## Author contributions

The authors contributed equally to the preparation of this article.

## Data Availability

The data that support the findings of this study are available in section Results and Discussion and Table 2 of this article.
